# Temperature-Dependent Reaction Kinetics of the Carbanions C_*n*_^–^ and C_*n*_H^–^ (*n* = 2 and 4) with H Atoms in a Cryogenic Ion Trap

**DOI:** 10.1021/acs.jpca.4c06626

**Published:** 2024-12-20

**Authors:** Christine Lochmann, Sruthi Purushu Melath, Michael Hauck, Robert Wild, Roland Wester

**Affiliations:** Institut für Ionenphysik und Angewandte Physik, Universität Innsbruck, Technikerstrasse 25/3, 6020 Innsbruck, Austria

## Abstract

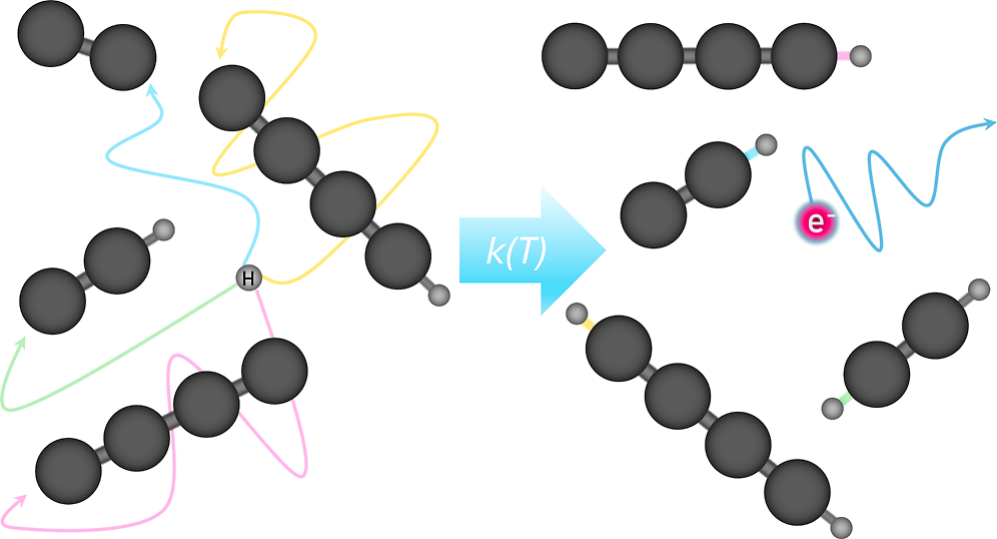

We report on the temperature-dependent reactions of the carbon-chain anions C_2_^–^ and C_4_^–^, as well as the hydrocarbons C_2_H^–^ and C_4_H^–^ with H atoms in the temperature regime between 8 and 296 K. The experiments have been carried out in a temperature-variable radiofrequency multipole ion trap. From the measured kinetics, we have derived reaction rate coefficients that are constant for all considered systems in the measured temperature regime. For the C_2_^–^, C_4_^–^, and C_4_H^–^ anions, the values are about a factor of 2 smaller than the Langevin capture rate coefficient, while for C_2_H^–^, the measured value agrees with the Langevin value. No theoretical calculations are available at present to explain this. All rate coefficients are in good agreement with previous measurements at room temperature.

## Introduction

Over 300 molecules have been identified in space so far in various different regions, most of them being neutral and positively charged species. A major constituent of these molecules is carbon chains, which are abundant and have been found in many molecular clouds.^1^ It is suspected that 10% to 25% of the space carbon is contained in the form of carbon chains or polycyclic aromatic hydrocarbons (PAHs).^2,3^ Interestingly, all eight of the confirmed negatively charged species are carbon-chain molecules and therefore are of special interest regarding their role in the formation of carbon chains and PAHs. Models show that after 10^5^ years, most of the negative charge in dense molecular clouds is bound to anions rather than free electrons.^4^ Although confirmation of their existence in the ISM is still missing, bare carbon-chain anions have been proposed to be present in the ISM due to the high abundance of neutral carbon chains.^5,6^ Naturally, the formation and destruction mechanisms of this class of anions are of great interest. For example, C_4_H^–^ and C_5_H^–^ have been shown to contribute to the formation of HC_5_N.^1^ The formation of the carbon-chain anions is expected to be mostly by radiative electron attachment,^7−9^ but another relevant pathway may be proton transfer,^9−11^ such as for the reaction H^–^ + C_2_H_2_ → C_2_H^–^ + H_2_.

In interstellar chemistry, hydrogen, carbon, nitrogen, and oxygen are the most prevalent species, but collisions with hydrogen are by far the most common due to its high abundance ratio. For this reason, reactions with atomic H are expected to be the dominant destruction mechanism for molecular anions under dense cloud conditions,^12,13^ while in photon-dominated regions, photodetachment is the major destruction channel.^14^ Here, we focus on short carbon chains and their hydrogen chemistry. Work at room temperature has been performed for C_*n*_^–^ and C_*n*_H^–^ (*n* = 2, 4–10) in SIFT tube measurements, showing reactions with atomic hydrogen via associative electron detachment^15,16^
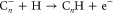
1
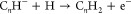
2Energetics calculations confirmed that exothermicities for association reactions also remained high with increases in chain length but were only experimentally observed for chain lengths with *n* ≥ 7, due to a decrease in the associative detachment rate with chain length. Reactions with H_2_ were found not to occur.^16,17^ The reactions with atomic hydrogen may have an appreciable temperature dependence, as we have recently observed for the reactions of the cyano anions CN^–^ and C_3_N^–^ with H atoms.^18^ Given the temperature range of molecular clouds (10–100 K), these reactions should be investigated in this temperature regime to ensure accurate rate coefficients are provided for modeling the chemistry in the ISM.

In this contribution, we report the reaction rate coefficients of C_2_^–^, C_4_^–^, C_2_H^–^, and C_4_H^–^ with atomic hydrogen in the temperature regime from 8 to 296 K and present the calibration of the hydrogen atom density through the reaction of chlorine anions with H.

## Methods

### Ion Trap Setup

**Figure 1:**
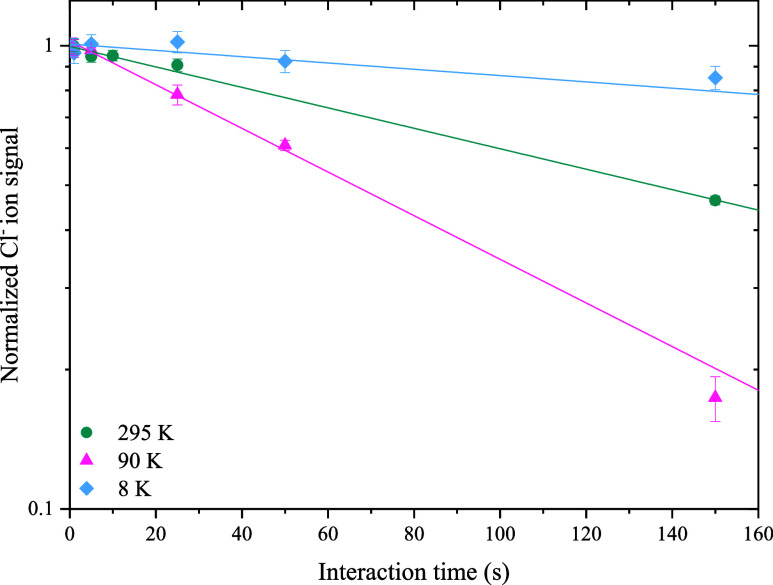
Chlorine ion signal as a function of interaction time with H atoms at three different trap temperatures.

The reaction studies are carried out in a cryogenic 16-pole radio frequency ion wire trap, which has been described in detail elsewhere.^19,20^ The ions are produced in a plasma discharge source through dissociative electron attachment. For the production of the carbanions, a mixture of 5% acetylene and 5% CO_2_ in 90% argon is used, while for the Cl^–^ ion, methyl chloride (CH_3_Cl) seeded in argon is used. The ions are extracted from the source region via a Wiley–McLaren time-of-flight mass spectrometer into the ion trap. The trap is mounted on a helium cryostat which allows for cooling down to 8 K. Additionally, through heating elements on the trap housing, temperature variation between 8 K and room temperature is possible. Due to the interaction with helium buffer gas, the ions are cooled down to the trap temperature, although at lower temperature, a slight deviation between buffer gas temperature and ion temperature is to be expected due to imperfections in the trapping fields.^20,21^ Finally, the ion signal is measured destructively on an MCP detector after variation of the interaction time between ions and H atoms. Examples of the ion signal after interaction of Cl^–^ with H taken at 295 K, 90 K, and 8 K are shown in Figure 1. The solid lines represent exponential decay fits to determine ion loss rate λ_loss_.

### Hydrogen Atom Effusive Beam Source

**Figure 2:**
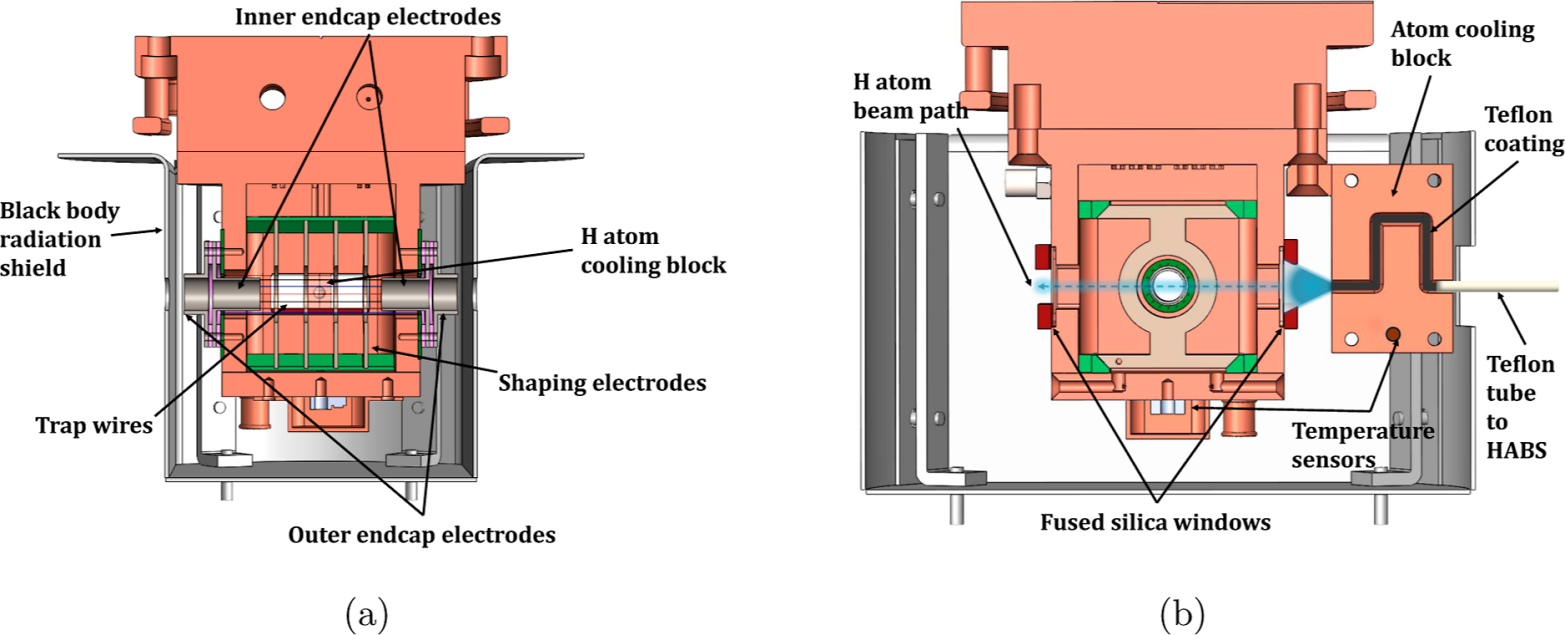
(a) Radial cross section of the trap housing. (b) Axial view of the trap housing, including a cross section of the atom cooling block and the presumed beam path (blue) into and out of the trap.

The hydrogen atoms are produced in a commercially available hydrogen atom beam source (HABS by MBE components) by thermal cracking of molecular hydrogen over a tungsten filament, operated at approximately 2000 °C. A Teflon tube guides the neutral beam into a copper block, which is thermally connected to the trap housing, to ensure the atoms are cooled to the same temperature as the trap. Cross sections of the trap setup are depicted in Figure 2.

The copper block consists of two separate plates into which a round U-shaped path has been drilled. A cross section of the block is depicted in Figure 2b. The path has a length of 57 mm and is coated with Teflon, to decrease the probability of the recombination of the H atoms to H_2_. The temperatures of the block and trap are monitored with silicon diode temperature sensors, placed on the bottom of the block and trap housing. The H atoms exit the block as an effusive beam through a round aperture, 3 mm in diameter. The aperture is at an 11 mm distance from the trap housing and is in line with the center of the trap, as can be seen in Figure 2a. In the axial direction, the trap is usually closed through two windows to increase the buffer gas density inside. For the purpose of this experiment, both full windows were replaced by two smaller windows on each side, leaving a 3 mm wide slit for entrance and exit of the neutral beam. As the aperture of the block is at about 1 cm away from the trap, the beam will somewhat diverge outward. Most of the atoms will therefore hit the trap and window surfaces, while a smaller amount will be able to pass through the trap as a continuous stream. We estimate from our calibration that under the present experimental conditions, a hydrogen atom density of approximately 10^6^ to 10^7^ cm^–3^, as well as an H_2_ density of about 10^11^ cm^–3^, is to be expected inside the trap.

### Hydrogen Atom Calibration

The hydrogen atom density inside the trap cannot be monitored directly from pressure measurements inside the trap housing due to the fast recombination to H_2_, which will lead to only H_2_ being detected by the pressure gauge. Commonly, chemical probing schemes are used to determine hydrogen number densities for calibration of the desired reaction rate coefficients. Only few reactions involving H atoms have been measured below room temperature. Three reactions with available temperature-dependent rate coefficients are CO_2_^+^ + H/H_2_,^22^ H^–^ + H,^23−25^ and D^–^ + H.^26,27^ However, none of these reactions is feasible for our current ion trap setup. In the case of CO_2_^+^ + H, the high amount of H_2_ in our neutral beam greatly hinders the detection of the reaction with H atoms. Trapping H^–^ would require significant changes to our RF trapping scheme and is therefore not a reasonable alternative. We instead use the reaction of hydrogen atoms with chlorine anions
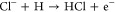
3

**Figure 3:**
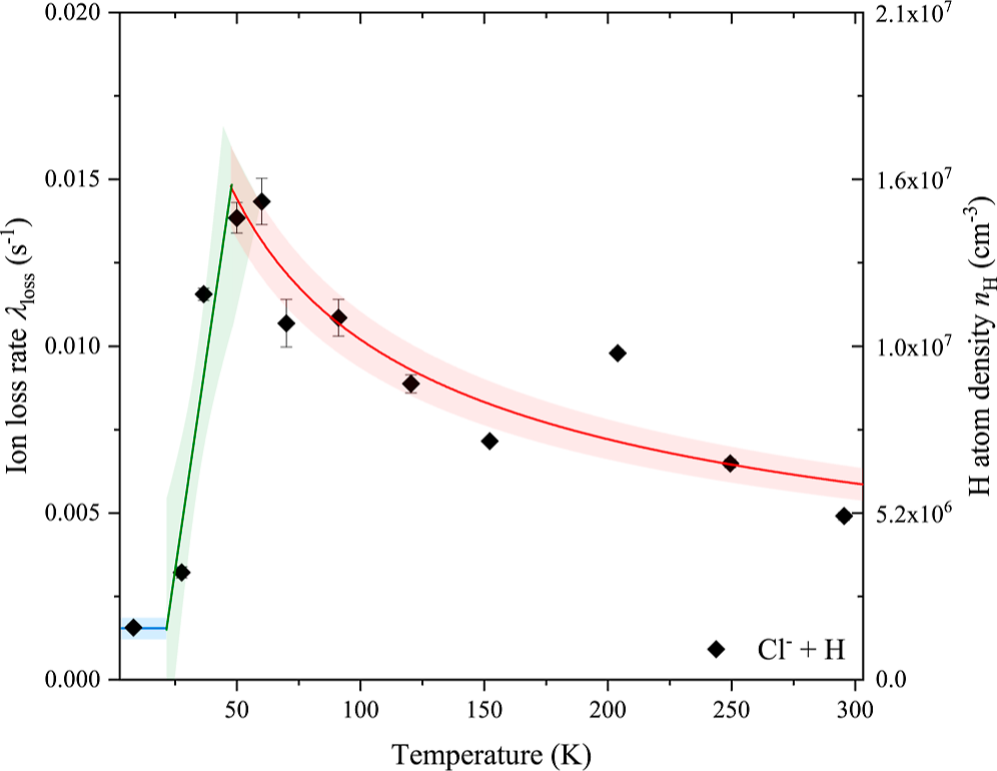
Chlorine ion loss rate and hydrogen atom density, assuming a constant rate coefficient of *k*_HCl_ as a function of trap temperature. The red line represents a fit (including the one sigma confidence interval) to a *T*^–1/2^ temperature dependence of the loss rate to guide the eye. This temperature dependence is due to the decreasing velocity of the H atom flux with decreasing temperature. Below 60 K, recombination of H to H_2_ leads to a linear decrease (green line) of the ion loss rate.

The associative detachment reaction of chloride with H has been extensively studied before, both experimentally as well as theoretically. Experimental reaction cross sections are available from crossed-beam measurements at collision energies between 0.1 and 20 eV,^28^ while thermal rate coefficients have been measured at room temperature using the flowing afterglow technique.^29−31^ The thermal measurements all yield rate coefficients of about 9 × 10^–10^ cm^3^/s and are in good agreement with each other within their respective uncertainties of about 20% to 50%. The more recent theoretical calculations,^32^ however, predict a higher rate coefficient of 1.4 × 10^–9^ cm^3^/s at 300 K. The reason for this discrepancy has not yet been definitively concluded but may lead to a deviation of the results for any rate coefficients which are calibrated using this reaction by a factor of about 40%. The most recent experimental value by Howard et al.^31^ of *k*_HCl_ = 9.6 × 10^–10^ cm^3^/s with an uncertainty of 20% will be used in the following to calculate the reaction rate coefficients.^18^Figure 3 shows the chlorine ion loss rate λ_loss_ as a function of temperature *T* in the range of 8 to 296 K. The plot also includes the corresponding H atom density *n*_*H*_, where *n*_H_ = λ_loss_/*k*_HCl_.

From Figure 3, we see that above 60 K, the ion loss rate is consistent with a *T*^–1/2^ dependence due the decreasing velocity of the effusive hydrogen beam with decreasing temperatures. Considering this temperature dependence, we assume that the Cl^–^ + H rate stays constant in this temperature regime. Below 60 K, we observe a decrease in the ion loss rate, which we have also observed previously.^18^ This decrease can be attributed to an increase in the recombination of H atoms to H_2_ on the cooling block surfaces. In the temperature regime between 10 and 30 K, no appreciable amount of H atoms can be observed due to such recombination, while below the freezing point of H_2_ (<10 K), the H atom flux increases again, and a constant factor is used for the calibration of this temperature point.

**Figure 4:**
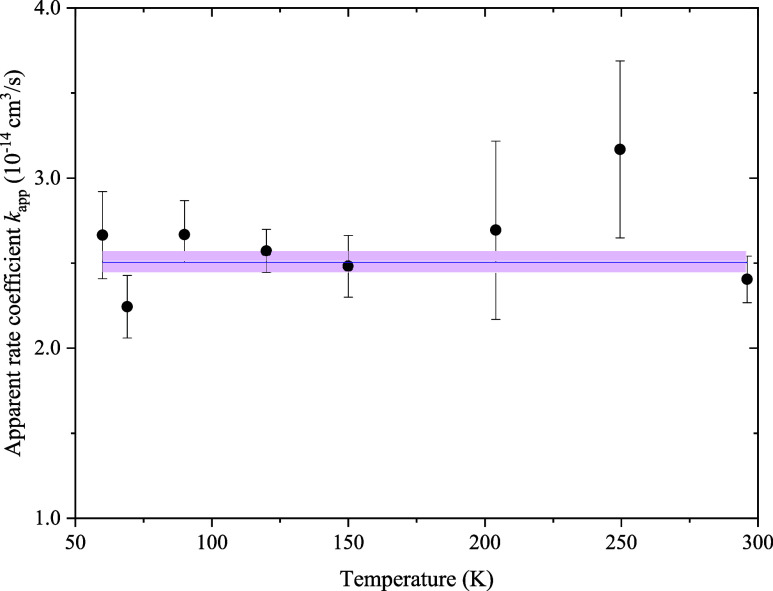
Apparent rate coefficients of Cl^–^ determined with the total hydrogen density (H + H_2_) in the trap.

To experimentally test the temperature independence of the Cl^–^ + H rate coefficient between 60 and 296 K, we additionally determined an apparent rate coefficient  as a function of the total hydrogen density *n*_H+H_2__ ≅ *n*_H_2__ inside the trap for each temperature. The density is calculated from the pressure measurement and the appropriate temperature scaling.^33^ The resulting *k*_app_, shown in Figure 4, are consistent with a constant (solid purple line, including the one sigma confidence interval). Since Cl^–^ does not react with H_2_ at such low densities,^21^ a temperature dependence of the rate coefficient would have to be balanced by an equal and opposite change in the fraction of atomic hydrogen coming from the H atom beam source. While possible, the low likelihood of such an occurrence strongly suggests a constant rate coefficient.

## Results and Discussion

**Figure 5:**
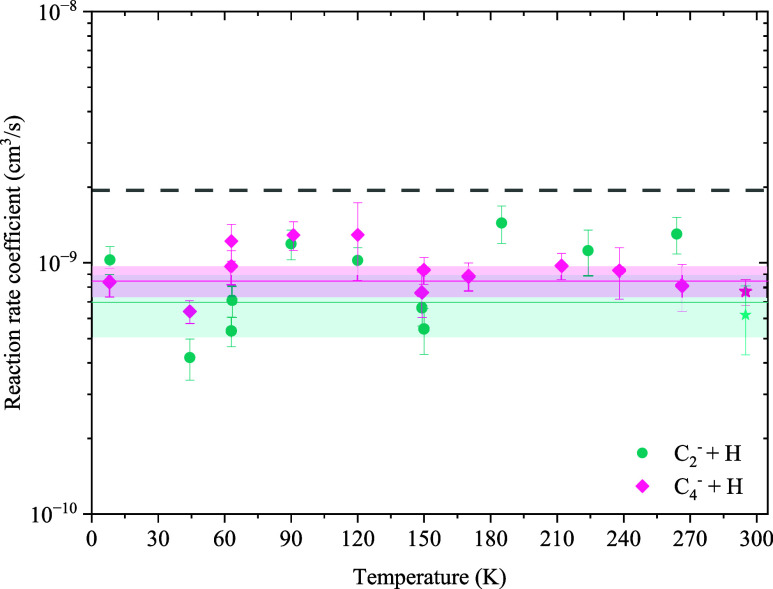
Temperature-dependent reaction rate coefficients of C_2_^–^ (green) and C_4_^–^ (pink) with H atoms. The dashed line corresponds to the Langevin capture rate coefficient. The stars are the rate coefficients determined by Barckholtz et al. at 298 K.^15^ The solid lines represent a constant fit throughout the indicated temperature range to determine an average rate coefficient.

**Figure 6:**
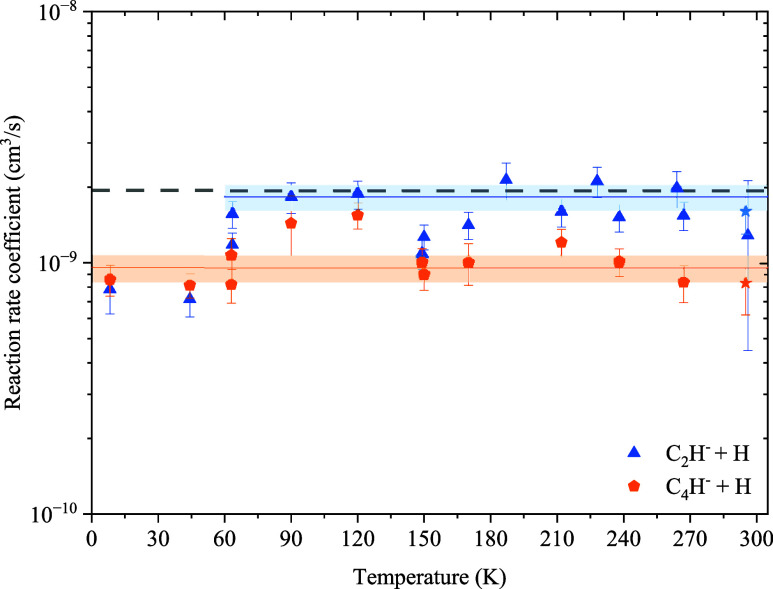
Temperature-dependent reaction rate coefficients of C_2_H^–^ (blue) and C_4_H^–^ (orange) with H atoms. The dashed line represents the Langevin capture rate coefficient. The stars are the rate coefficients determined by Barckholtz et al. at 298 K.^15^ The solid lines are constant fits in the indicated temperature range to determine an average reaction rate coefficient.

Figures 5 and 6 show the reaction rate coefficients for the reaction of the C_*n*_^–^ + H and C_*n*_H^–^ + H systems, respectively. The error bars on the data include the statistical uncertainties from the fits of the loss rate and calibration functions, as well as fluctuations of the pressure measurements. At all temperatures, separate background measurements of the ion loss rates with the He buffer gas and a mixture of He and H_2_ have been taken and subtracted from the foreground measurements with H atoms. However, since the trap cannot actively be cryocooled at temperatures above 150 K, changes in the background gas composition during these measurements, which might affect the background ion loss rate significantly, cannot be ruled out and are difficult to reflect in the error bars. The major background gas constituents in our trap are N_2_, O_2_, and H_2_O. While reactions with nitrogen and oxygen are negligible,^34^ reactions with water might considerably contribute to the background loss rate. Consequently, we attribute the larger scatter of the data points to fluctuations in the background loss rates, which were especially pronounced in the case of C_2_^–^. Note that no reactions with H_2_ can be observed for any of the studied anions at the present hydrogen densities, which are in the range of 10^11^ to 10^12^ cm^–3^.^17^

None of the considered systems show clear evidence of a change in the reaction rate coefficient with temperature, which is in stark contrast to our previous results from the measurement of the cyano anions CN^–^ and C_3_N^–^ with H atoms, which show an inverse temperature dependence.^18^ This difference points to different curve crossing dynamics from the anionic to the neutral potential energy surfaces but cannot be explained at present. Constant fits to the present data were performed to determine the average reaction rate coefficients in the measured temperature regime. The resulting rate coefficients are summarized in Table 1 and shown as solid lines, including a one sigma confidence interval, in Figures 5 and 6. In the case of the C_2_H^–^ + H system, the data at 45 and 8 K have been excluded from the fit, as here, a significantly lower rate is observed, which does not agree with the model of a temperature-independent rate coefficient. Including these points in the fit would result in a 30% lower rate coefficient.

**Table 1:** Comparison of the Reaction Rate Coefficients for the Reactions of the C_*n*_^–^ and C_*n*_H^–^ Anions with H Atoms Determined in This Paper, the 298 K Values from Barckholtz et al.,^15^ and the Langevin Capture Rate Coefficients^a^

anion	this paper	Barckholtz et al. at 298 K^15^	Langevin
C_2_^–^	7.0 (9)	7.7 (9)	19.4
C_4_^–^	8.5 (5)	6.2 (1.9)	19.2
C_2_H^–^	18 (1)	16 (3)	19.4
C_4_H^–^	9.6 (5)	8.3 (2.1)	19.3

a All rate coefficients are given in units of 10^–10^ cm^3^/s, the values given in the brackets represent the one sigma statistical uncertainties.

A comparison of the present results and the results from the 298 K rate coefficients taken by Barckholtz et al.^15^ is shown in Table 1. Although the present results show slightly higher values, there is excellent agreement between the measurements, considering the statistical uncertainties of both. Additionally, the Langevin capture rate coefficients are also presented. Note that if the ions’ dipole and quadrupole moments are also considered in the capture collision rate coefficients,^35^ then the coefficients would increase substantially and a positive temperature dependence would be expected. With the exception of C_2_H^–^, all reactions proceed at about half of the Langevin rate, which was also observed by Barckholtz et al.^15^

C_2_H^–^ attachment of the H atom on the carbon end as well as on the hydrogen-terminated end may lead to associative detachment, which may explain the near Langevin rate coefficient. In this case, attachment to the H-terminated end and stabilization of the intermediate vinylidene anion through a rearrangement of one of the H atoms to the C-terminated end may occur, followed by the formation of neutral acetylene and a free electron. The probability for such a rearrangement should decrease with increasing length of the chain, which may explain the difference in the reaction efficiency between C_2_H^–^ and C_4_H^–^ species. It may be also assumed that the vinylidene–acetylene isomerization is hindered at low temperatures by a barrier in the potential energy landscape, which can explain the decrease in rate coefficient at 45 K and below by about a factor of 2. In the case of the bare carbon-chain anions, a tentative explanation for the reduced reaction efficiency compared to the Langevin capture limit could be a difference in the reactivity with respect to the angle of the approaching H atom, i.e., that a T-shaped approach is less favorable than a colinear approach. However, detailed ab initio calculations are necessary to understand these different rate coefficients.

## Conclusions

In this contribution, we present the temperature-dependent reaction rate coefficients of the carbon-chain anions C_*n*_^–^ and hydrocarbon anions C_*n*_H^–^ (with *n* = 2 and 4) with atomic hydrogen using a cryogenic 16-pole wire trap. Three of the four studied reactions show no evidence of a temperature dependence; only for C_2_H^–^ is a drop of the rate coefficient observed at low temperatures. This is qualitatively discussed by comparing different angles of approach of the incoming hydrogen atom. A comparison with the available room temperature data shows excellent agreement in all cases.

The associative detachment of hydrogen atoms is an important destruction mechanism for anions in the interstellar space, especially in the environment of cold molecular clouds.^4,10^ The present results and our previous results on the reactions of H atoms with cyano anions^18^ show the need to confirm the reaction rate coefficients in the temperature regimes prevalent in the regions of space where anions contribute to the chemical pathways.
